# Lived Experience of Caregivers of Lung Transplant Recipients in Korea

**DOI:** 10.3390/healthcare13131608

**Published:** 2025-07-04

**Authors:** Haeng-Mi Son, Kyoungok Min, Younghui Hwang

**Affiliations:** 1 Department of Nursing, College of Medicine, University of Ulsan, 93 Daehak-ro, Nam-gu, Ulsan 44610, Republic of Korea; sonhm@ulsan.ac.kr; 2 Department of Transplant Center, Seoul National University Hospital, 101 Daehak-ro, Jongno-gu, Seoul 03080, Republic of Korea; ismelin@hanafos.com; 3 Department of Nursing Science, College of Nursing, Research Institute of Nursing Science, Chungbuk National University, 1 Chungdae-ro, Seowon-gu, Cheongju 28644, Republic of Korea

**Keywords:** caregivers, lung transplantation, qualitative research

## Abstract

**Background/Objectives:** This study aimed to explore the underlying meaning and structure of the experiences of caregivers with lung transplant recipients using phenomenological research methods. **Methods**: Data were collected between February 2020 and December 2021 via in-depth individual interviews with nine caregivers of lung transplant recipients. The meaning of the participants’ experiences was analyzed using Colaizzi’s phenomenological analysis to ensure methodological rigor. Researchers minimized bias through reflexivity and member checking, and the study adhered to ethical standards to ensure trustworthiness. **Results:** Participants cared for patients who had not fully crossed the threshold of death without giving up hope for a cure. They did not avoid caregiving as a responsibility to their families but accepted it as their responsibility. The lives of the participants became increasingly immersed as they witnessed the process of the patient’s illness and gained insights into patience and gratitude through the caregiving experience. **Conclusions:** This study’s findings can help assess the needs of lung transplant recipients and their caregivers and guide interventions that address their reciprocal relationship. It also emphasizes the importance of ongoing education and expanded social care services to reduce caregiver stress and burden.

## 1. Introduction

Lung transplantation is recognized as an effective treatment for improving the life expectancy and quality of life in patients with end-stage lung disease [1,2]. However, to prevent graft rejection and manage post-transplant complications, lifelong immunosuppressive therapy and careful medical monitoring are essential [3]. Caregiver support is a vital component of post-transplant care, significantly influencing the incidence of chronic rejection and the long-term survival of lung transplant recipients [4].

Despite its benefits, lung transplantation demonstrates lower long-term graft survival rates and poorer patient prognosis compared with other organ transplants such as kidney, liver, and heart transplants [5]. The lives of caregivers of lung transplant recipients may be adversely affected by these low graft survival rates and poor prognoses. These caregivers experience anxiety, fear, frustration, uncertainty, and extreme stress as they witness the changing conditions of their patients [6]. Therefore, caregivers of lung transplant recipients may undergo more significant life changes than those of other organ transplant recipients. However, research on caregiving experiences specific to lung transplant caregivers remains limited. Identifying the experiences of caregivers is crucial for understanding the challenges they encounter and developing effective social support systems tailored to their specific needs.

Caregivers experience the disease trajectory alongside the lung transplant recipients, from the diagnosis of end-stage lung disease to the transplantation process [7]. They play an important role in assisting recipients with post-transplant care, support that is significant enough to influence the incidence of chronic rejection and long-term graft survival [4]. Thus, understanding the caregiving experiences of lung transplant caregivers may contribute to developing strategies aimed at reducing chronic rejection and improving the long-term survival rates in lung transplant recipients.

Caregiving experiences and the challenges faced by lung transplant caregivers may vary according to sociocultural context and racial background [7]. For example, Hispanic caregivers perceive the diagnosis of end-stage lung disease as the most significant shock, while White American caregivers report experiencing persistent depression and anger throughout the disease trajectory. In contrast, African American caregivers report experiencing greater financial difficulties [7]. The experiences of lung transplant caregivers may not be adequately captured through objective methods, as the depth and intensity of these experiences can vary significantly across different sociocultural contexts. Phenomenology is a qualitative research method that aims to describe the “inner consciousness” of participants, interpret the internal meanings derived from experiences, and systematically elucidate the essence of those experiences [8]. Therefore, qualitative research is essential for describing and understanding the vivid and subjective experiences of caregivers. This study aimed to explore the subjective and personal experiences of lung transplant caregivers in Korea and gain an in-depth understanding of the meanings they attribute to these experiences through a phenomenological qualitative research approach.

By exploring the structural meaning and essence of caregiving experiences, this study seeks to provide an in-depth understanding of the lived experiences of caregivers. Such an understanding could contribute to developing future intervention programs designed to support the caregivers of lung transplant recipients.

The purpose of this study is to explore the meaning and essence of caregiving experiences among caregivers of lung transplant recipients using a phenomenological qualitative research approach. The research question guiding this study is ‘What are the experiences of caregivers of lung transplant recipients, and what are the structural meanings and essence of these experiences?’.

## 2. Materials and Methods

### 2.1. Study Design

This qualitative study employed a descriptive phenomenological approach to understand the essence of the lived experiences of caregivers of lung transplant recipients. Descriptive phenomenology, originating from Husserl’s eidetic concept, aims to discover, explore, and describe uncensored phenomena. Husserl sought to understand the foundation of knowledge from which all things arise as a necessary condition for experience [9]. The phenomenological method is appropriate, as it enables an in-depth exploration of human experiences within the context of everyday life [8]. Accordingly, the research question guiding this study is ‘What is the essence of the caregiving experience for caregivers of lung transplant recipients?’.

### 2.2. Participants

The participants in this study were caregivers of lung transplant recipients registered at the Organ Transplant Center of a university hospital. The specific inclusion criteria were as follows. First, participants had to be adults aged ≥18 years who were capable of freely expressing their experiences. Second, only primary caregivers of the lung transplant recipients were included. In cases where multiple family members, such as spouses, parents, children, or siblings, shared caregiving responsibilities, the primary caregiver was identified as the individual who was mainly responsible for daily care, attending outpatient appointments, and assisting during hospital stays. Third, only individuals who voluntarily consented to participate in the study were included. To ensure diversity in demographic characteristics, such as sex, age, marital status, and employment status, purposive sampling was used. The researchers explained the purpose and methodology of the study to the primary caregivers and obtained their written informed consent to participate. Interviews were conducted until data saturation was reached, that is, when no new themes emerged and the content was repeated. Thus, nine participants were included in the final analysis.

### 2.3. Data Collection

Data were collected through one-on-one in-depth interviews. The primary interview questions included ‘Can you describe how your daily life changed while caring for the patient before and after the transplant?’; ‘What were the most challenging aspects of caregiving before and after the transplant?’, and ‘What types of support or resources were helpful to you during caregiving before and after the transplant?’. Each interview began with a light, casual conversation to establish rapport between the researcher and the participants before transitioning to the formal questions. Interviews were conducted in locations where the participants felt most comfortable such as a nearby café or the hospital room. Each session lasted between 1 and 2 h and was audio recorded. Each participant took part in a single, face-to-face interview. During the interviews, the researchers observed the nonverbal behaviors of the participants and their reactions. Immediately after each interview, they documented any significant nonverbal expressions, observations, and reflections. Data collection was conducted between February 2020 and December 2021 and carried out until data saturation occurred, when the same content was repeated in the participants’ interviews, and no additional information was provided for new understanding.

Following each interview, a research assistant transcribed the audio recordings verbatim. The researchers then listened to the recordings repeatedly to ensure accuracy in the transcriptions. Data collection and analysis were conducted simultaneously.

### 2.4. Data Analysis

Two primary researchers (Son and Hwang) analyzed the data using Colaizzi’s phenomenological method [10]. To gain a comprehensive understanding of the caregiving experiences of lung transplant caregivers, the researchers focused on how these experiences influenced and transformed the lives of participants from the pre-transplant phase onward. To extract meaningful insights from the data, the researchers repeatedly read the interview transcripts, highlighted key phrases and sentences, and identified meaning units by assigning descriptive labels to the highlighted sections. Subsequently, redundant or overlapping meaning units were removed, while similar ones were grouped to form emergent meaning clusters. These meaning clusters were then reorganized into abstract and generalized statements in the researchers’ own words, resulting in the development of subthemes. A rigorous, iterative process was used to ensure consistency between the subthemes and the original statements. The researchers continuously refined their wording and examined the transcripts to ensure their interpretations remained true to the experiences of the participants. The final subthemes were then organized into overarching themes to accurately reflect the underlying structure of the caregiving experiences of the participants. To ensure the coherence and validity of the identified themes, the researchers repeatedly referred to the raw data, cross-checked it for consistency, and revised their interpretations as necessary. Finally, the fundamental structure of the caregiving experience was identified through phenomenological reduction. A comprehensive account of the lived experiences of caregivers was developed, which centered on the emergent themes. Throughout the research process, the researchers maintained reflective theoretical and methodological memos and facilitated regular communication to ensure balanced and unbiased data interpretation.

### 2.5. Researcher Reflection

The researchers have experience caring for transplant patients in organ transplant centers or internal medicine wards. Two of the researchers, professors from nursing departments, conducted multiple qualitative research studies as members of a qualitative research association. The third researcher is an organ transplant coordinator working at the organ transplant center.

Compared with other types of organ transplants, the number of patients receiving lung transplants remains relatively low, and lung transplantation has a poorer prognosis. Therefore, the researchers hypothesized that the lives of caregivers would be significantly affected by the poor clinical progression of lung transplant recipients. Recognizing the scarcity of related studies, the authors identified a clear need to conduct this research.

The researchers’ initial assumptions regarding the study were as follows: ‘Caregivers of lung transplant recipients experience severe psychological stress’, ‘Caregivers of lung transplant recipients desire to escape their situation’, and ‘Caregivers of lung transplant recipients hold negative perceptions of lung transplantation’. To prevent these assumptions from introducing bias into the study, the researchers maintained an epistemological distance from their preconceptions and ensured that these assumptions did not influence the data interpretation. Through repeated, immersive reading of the interview data while bracketing their professional experiences, they sought to authentically represent the participants’ experiences.

### 2.6. Ethical Considerations

This study was reviewed and approved by the Institutional Review Board (IRB) of Seoul National University Hospital (IRB No. 2001-133-1096). The researchers provided each participant with an informed consent document outlining the purpose of the study. This included information on the research process, potential benefits and risks, data confidentiality, audio recording of interviews, voluntary participation, and the right to withdraw at any time. Written informed consent was obtained from all participants.

The researchers ensured that all audio recordings and transcribed interview data were securely stored on a password-protected personal computer and used exclusively for research purposes before being properly discarded. Participants were informed about these data protection measures. Additionally, a small token of appreciation was given to each participant for their involvement in the study.

### 2.7. Trustworthiness of the Study

To ensure the trustworthiness of the study, the criteria proposed by Lincoln and Guba [11] were applied. To establish credibility (truth value), purposive sampling was employed, considering the diverse demographic characteristics of the participants. The recorded interviews were transcribed verbatim, and the researchers verified the accuracy of the transcripts by listening repeatedly to the recordings. Based on the empirical data of the participants, significant statements, meaning units, subthemes, and themes were derived. Relevant participant quotes were included to enhance credibility. Additionally, member checking was conducted by sharing the research findings with two participants to confirm that their experiences were accurately represented and free from distortion. Their feedback included responses such as ’That really clarified what I was saying’, ‘That’s exactly how I felt (yes).’, and ‘I still do that now’.

To establish applicability, diverse demographic characteristics such as sex, age, marital status, and employment status were considered in the participant selection. The characteristics of the participants and the data collection process were described, enabling readers to assess the applicability of the findings to similar contexts and situations.

For consistency, Colaizzi’s [10] data analysis procedure was rigorously followed and described in detail to ensure traceability for readers. The researchers extracted significant statements from the transcribed data, organized them into meaning units, and identified subthemes, which were further synthesized into themes. Throughout this process, the original data were continuously reviewed to ensure analytical appropriateness and comprehensively capture the essence of the experiences of the caregivers, thereby reinforcing the validity of the findings.

To maintain neutrality, the researchers adopted a nonjudgmental stance during the interviews, actively listening to the participants’ narratives while bracketing their own preconceived notions and professional assumptions. The research team maintained reflexivity throughout the study by engaging in ongoing discussions and building consensus during the interpretation of the findings. Two primary researchers (Son and Hwang) conducted the analysis, which was verified by a co-researcher (Min). Final results were established through agreement among the three researchers.

To enhance rigor, the research process and findings were reviewed by an expert in qualitative research. Additionally, to ensure transparency, the research process was evaluated using the Consolidated Criteria for Reporting Qualitative Research (COREQ) [12].

## 3. Results

This study included nine participants—six females and three males. Regarding their relationship with the lung transplant recipients, six were spouses, two were either the mother or sister of the patient, and one was a sister-in-law. The participants had a mean age of 64.14 ± 7.06 years, ranging from 53 to 77 years. The duration of caregiving ranged from 11 months to 20 years prior to lung transplantation, and from 2 months to 3 years and 6 months post-transplantation.

Twelve subthemes were identified and subsequently integrated into four themes that represented abstract and comprehensive meanings (Table 1). Participants shared their experiences of caring for lung transplant recipients who had narrowly survived a life-threatening illness but had not fully recovered. When the patients required assistance, caregivers prioritized their needs, providing unwavering support and demonstrating selfless and unconditional love in their caregiving roles.

### 3.1. Caring for a Patient Who Has Not Fully Crossed the Threshold of Death

Before transplantation, lung transplant recipients were merely waiting for death, with no viable treatment options remaining. Participants described the helplessness of watching their loved ones suffer from severe dyspnea, facing the imminent risk of death due to end-stage lung disease. Consequently, the participants and patients clung to the hope of a lung transplant as their sole chance of survival.

After the transplant, the participants were relieved to witness some improvement in the health of the recipients and hoped that they would live long, fulfilling their purposes. However, this relief was accompanied by persistent anxiety, as the caregivers remained acutely aware that even a minor symptom, such as a cough, could signal a life-threatening lung infection. This constant vigilance and fear of sudden decline imposed a heavy emotional burden on the caregivers. Every minor change in the health of the patients triggered waves of uncertainty and distress. The themes reflecting this experience included watching a loved one in agony at the brink of death, the pendulum-swinging uncertainty of hope, and holding on for the survival of the patient.

#### 3.1.1. Watching a Loved One in Agony at the Brink of Death

Before the lung transplant, patients were often bedridden, unable to move due to severe shortness of breath, and entirely dependent on supplemental oxygen. During this period, the caregivers frequently experienced profound helplessness, unable to provide relief. The patients, living daily in a state of physical decline, were described as awaiting death, as even the medical team had lost hope. The fear that the patient could die at any moment due to extreme respiratory distress caused profound emotional suffering. Lung transplantation was the sole means of saving the life of the patient.

*“Before the transplant, the patient was practically like a lifeless body. The patient was alive, yet unable to move, just lying there while I had to keep going.”*

(Participant 4)

*“The fear of losing him… No one can truly understand that kind of fear.”*

(Participant 3)

#### 3.1.2. Pendulum of Hope Swinging Through Uncertainty

The lung transplant was perceived by the caregivers as a stroke of fate. As the caregivers observed the patient gradually regain basic functions, they felt reassured that the transplant was the only path to survival. However, complications soon emerged as the patients began to develop health-related complications that limited their independence, requiring continued caregiver support. Recurrent lung infections often lead to prolonged hospitalization or frequent readmission. The caregivers realized that lung transplantation did not guarantee a full recovery. As the health of the patients declined further, resembling that of the pre-transplant state, the caregivers experienced growing anxiety and despair, fearing the possibility of death.

*“(During hospitalization after the transplant), there was a time when the condition of my husband deteriorated significantly. At one point… I even thought, ‘so this is how a person dies, because if he can’t breathe, he will die.”*

(Participant 6)

*“I thought a lung transplant would be a cure, that it would save their life. But it was not like that. Later, I searched the Internet again and realized that the life expectancy after transplantation was not as long as I had hoped.”*

(Participant 8)

#### 3.1.3. Holding on for the Survival of the Patient

Caregivers deeply wished for patients who had endured prolonged hospitalizations or multiple readmissions after lung transplantation to overcome their suffering and continue living. Watching their loved ones face ongoing battles with illness evoked profound sorrow, and they genuinely wanted to help them regain their health and happiness. Therefore, the caregivers could not turn away from patients who were willing to live. The sense of family provided the caregivers and patients with comfort and strength, making it impossible for them to give up.

*“They want to live. Therefore, I cannot let go. I cannot give up… because they want to live.”*

(Participant 2)

*“They are so young, and it is just heartbreaking. My heart aches, it stings… I cannot even put it into words.”*

(Participant 3)

### 3.2. Inevitability of Care

The caregivers expressed a strong sense of responsibility rooted in their roles as family members. They believed that their circumstances and emotional closeness made them the most suitable caregivers. Therefore, they naturally assumed the roles of primary caregivers. Despite the challenges they encountered, they accepted caregiving as a duty. The themes included ‘Family care as a natural responsibility’, ‘The suitable person for patient care’, and ‘Making every effort to care’.

#### 3.2.1. Family Care as a Natural Responsibility

The caregivers viewed caring for patients as a natural duty of family members. Particularly when the caregiver was a parent, they viewed taking care of their sick child as an unquestionable responsibility and therefore devoted themselves entirely to it. Some caregivers quit their jobs or changed their workplaces to fully dedicate themselves to caring for the patients.

*“I just have to do it because we are family. If I can do it, I should. That is how I feel.”*

(Participant 1)

*“I gave birth to my child, so that is a fact, and with that comes responsibility. I have a duty to do what I can for them.”*

(Participant 3)

#### 3.2.2. Suitable Person for Patient Care

Caregivers believed that they were the most suitable individuals to care for the patients because they had the necessary time and financial resources. They expressed confidence in their ability to provide proper care. The primary caregivers assisted whenever needed, regardless of time or place, throughout the illness. When the patients felt comfortable with and relied on them, the caregivers developed emotional bonds with them, often leading them to voluntarily assume the primary caregiver role.

*“It is not like I have a job—I am just a homemaker… If I were working, would I be able to do this? No, I could not. However, since I am at home and I have the flexibility to move around, I take on the role.”*

(Participant 4)

*“At that time, I was originally working. Nonetheless, I did not renew my contract and started taking care of her. I think she relied on me a lot. Her mother lived far away, and she was running a business. Therefore, there was no one else to depend on. That was why she leaned on me so much. Even now, I feel like she still depends on me a lot.”*

(Participant 1)

#### 3.2.3. Making Every Effort to Care

The caregivers devoted themselves wholeheartedly to caregiving, driven by a deep desire to avoid future regrets if the patient were to pass away. Their efforts included meticulous attention to medical routines such as the regular administration of immunosuppressants, implementing dietary management, and maintaining a clean environment through frequent disinfection and the laundering of bed linens to prevent infections. Among the various emotions experienced throughout their caregiving journey—joy, sorrow, love, and pain—feelings of guilt and pity prevailed. These emotions fueled their unwavering commitment to do everything they could for the patient.

*“(When I) listen to longtime caregivers, I realize that many continue caring out of guilt. Most seem to feel that way… If they do not do everything they can, they feel like they will not be able to live with themselves after the patient is gone. Therefore, they try to do their best to avoid any regrets once the patient passes away.”*

(Participant 2)

*“For foods that have not been cooked, I only allow them to taste a small amount—just a bite or two. Other than that, I strictly prohibit them from eating anything else and have carefully planned their diet.”*

(Participant 8)

### 3.3. My Life Is Buried in the Life of a Patient

The daily lives of caregivers gradually became entirely intertwined with those of the patients. Personal routines were progressively replaced by the demands of caregiving, often taking precedence over their needs and even those of other family members. The themes included ‘Prioritization of the patient over my daily life’, ‘Patient-centered priority over other family members’, and ‘My becoming the companion in the disease journey of patients’.

#### 3.3.1. Prioritizing the Patient over My Daily Life

To care for the patients, the participants gave up certain aspects of their own lives. A few left their jobs to devote themselves entirely to caregiving, whereas others chose employment that allowed them to balance their work and caregiving responsibilities. Despite undergoing radiation therapy for cancer, one participant prioritized patient care over her treatment. Participants who provided 24-h care in the hospital experienced significant physical exhaustion but continued to place the health of the patients above their own. They consciously avoided social gatherings or activities aimed at building relationships, choosing instead to remain by the side of the patients.

*“If the patient just gets better, then… well, I mean, I also like exercising, meeting people, and going out. However, for now, this (caregiving) comes first.”*

(Participant 1)

*“I had been working in hospital cleaning for over 10 years, but when the patient became very ill, I quit. Then, I started working at places such as restaurants. I would bring the patient with me, seat him there, and work from 7 p.m. to 11:30 p.m.”*

(Participant 7)

#### 3.3.2. Patient-Centered Priority over Other Family Members

As the participants became increasingly absorbed in caregiving, their ability to fulfill other family responsibilities diminished. When the patients were hospitalized, the caregivers often stayed overnight or returned home late at night, making it challenging to manage their household responsibilities. A deep sense of guilt lingered, particularly regarding their children—those still needing their maternal attention such as those preparing for college entrance exams or returning home from work. One participant, who was caring for her sister, shared that her husband understood and supported her exhaustion from caregiving; however, she still felt unease and remained deeply conscious of his feelings on how her absence might be affecting him.

*“I also feel sorry for my son because he has been eating dinner alone all this time. When he came home, no one was there… Sometimes, I even left without giving him breakfast.”*

(Participant 2)

*“Since I was at the hospital, there were times when the patient was not doing well, and I got home really late. During those times—things such as preparing meals for my husband and the kids—I just could not manage them. Back then, when the patient was really sick, my son was preparing for his college entrance exams. Later, when the patient underwent the transplant, my son had failed the exam and was preparing to take it again. During those periods, I could not help but feel self-conscious around my children and my husband.”*

(Participant 1)

#### 3.3.3. Becoming the Companion in the Disease Journey of the Patient

Participants empathized with and embraced the physical pain of the patients and their emotional struggles and suffering, responding sensitively to the words and behaviors of their patients. Over time, they became the individuals who best understood the patients. Without even realizing or intending it, the participant became a companion in the lives of patients, having walked alongside them through every stage of the illness from before the lung transplant to the present.

*“(When the patient is admitted) If I am at home, honestly, I worry more about the patient. When I see her in the hospital with my own eyes, I know she is okay because I can check on her. However, when I am at home and not checking, I worry about what she might be doing right now. I even texted her on KakaoTalk, and if I do not get an immediate reply, I start wondering if she is in pain.”*

(Participant 1)

### 3.4. Insights Gained from Patient Care

Through the caregiving experience, participants realized the essence of emotional acceptance and letting go despite their hardships. As they made efforts to view the condition of the patients more positively, their daily lives began to have a more optimistic outlook. Furthermore, as the participants perceived that patients and other family members acknowledged their efforts, they developed a renewed sense of self-worth and fulfillment in their caregiving role. The themes within this category included ‘Enduring and letting go’, ‘Sense of fulfillment’, and ‘Feeling of gratitude’.

#### 3.4.1. Enduring and Letting Go

Caring for a lung transplant recipient was physically and emotionally demanding, but the participants endured it with patience and perseverance. Participants blamed themselves and experienced stress when their condition worsened, questioning whether this was due to inadequate care on their part. They often felt a sense of urgency, hoping for the rapid recovery of the patients and an end to the difficult situation; however, they realized that such impatience was not helpful in healing. Consequently, they practiced letting go of expectations and accepting challenges with a more open and patient-oriented mindset.

*“Being impatient does not change anything. I mean, does anything get better just because I am impatient? If anything, the patient becomes even more anxious. I just hope each day brings improvement. However, showing that impatience on the outside—what good does that do? It does not help at all.”*

(Participant 3)

*“I think it is about accepting things as they are—like, ‘this is as far as we can go.’ That is how I see it. Therefore, I believe other caregivers should try to ease their minds a bit. There is no need to get so anxious and worked up.”*

(Participant 4)

#### 3.4.2. Sense of Fulfillment

Despite the many challenges of caregiving, the participants experienced a sense of fulfillment because the patients relied on them, and their family members expressed trust in and gratitude for their efforts. Participants recognized their value through these affirmations, finding purpose and meaning in playing a vital role in their recovery.

*“I realized that I am really an important person to her, that she truly needs me. That gives me a sense of fulfillment. I am over 50 years old now and going through menopause—everyone at home is always saying they are sick, and the kids do not treat their mom the way they used to. However, here, I feel like I am really needed.”*

(Participant 1)

*“My mom says to me, ‘I am so thankful you are here. If you were not, who else would do all this?”*

(Participant 4)

#### 3.4.3. Feeling of Gratitude

As the participants gradually came to terms with their reality and released some of their emotional burdens, they became more emotionally available and reflective. This transformation fostered a deep sense of gratitude to the lung donors who gave the patients a new lease on life as well as to the medical staff who devoted themselves wholeheartedly to caring for the patients before and after the lung transplant. They also were thankful to other family members who shared their emotional labor and caregiving concerns.

*“I know I should not feel this overwhelmingly happy… but I really have to live with gratitude for the donor.”*

(Participant 4)

*“I am grateful that the doctors from various departments discussed the case thoroughly and worked together to make the lung transplant happen. The nurses were very kind and caring.”*

(Participant 3)

*“If I had done this alone, without help from other family members… it would have been almost impossible. I might not have been able to do it at all.”*

(Participant 1)

## 4. Discussion

Caregivers described the pre-transplant period as a time of profound distress, marked by caring for patients who barely sustained life through oxygen therapy. Even after the transplant, patients experienced anxiety and uncertainty regarding their condition. These findings align with those of previous studies, indicating that witnessing the progressive decline in the health of patients is a significant stress for caregivers [7] and that caregivers frequently experience anxiety and uncertainty in response to the health of patients [6]. Therefore, alleviating caregiver stress requires a deeper understanding of their needs. A prior study reported that caregivers of patients awaiting lung transplantation were most concerned with the prognosis of the patients after lung transplant [13], underscoring the essence of providing accurate and clear information from healthcare professionals regarding post-transplant outcomes. Healthcare providers should thus offer detailed explanations to caregivers about possible post-transplant scenarios before the procedure. Enhancing the caregivers’ understanding in this manner may potentially minimize their anxiety and uncertainty, ultimately mitigating caregiver stress after transplantation.

Lung transplant patients often describe their post-transplant life as a ‘second chance’, enduring challenges after the surgery because they recognize the value of the life they have regained [14]. Compared with kidney or liver transplants (median survival > 20 years), lung transplants show a significantly lower median survival (6.7–9.2 years) [15,16]. Over 50% of recipients develop chronic complications within five years post-transplantation, with frequent health deterioration leading to repeated hospitalization. Both patients and caregivers faced recurring mortality fears while maintaining hope for recovery and normalcy. Caregivers observed that lung transplant recipients endured significant physical and psychological pain and difficulties following surgery to protect the renewed life gained through transplantation. Caregivers continued to provide unwavering support, motivated by their awareness of the recipients’ strong desire to live. This study’s most significant finding is that patients’ determination to survive became the driving force that enabled caregivers to persist in their care, while the caregivers’ dedicated support may positively influence patient outcomes. This reveals that the relationship between lung transplant recipients and their caregivers is not a one-sided provision, but rather a reciprocal relationship where both parties draw strength and encouragement from each other. Additionally, this study highlights the importance of providing end-of-life education for both recipients and caregivers who live with the constant fear and uncertainty surrounding death.

The theme identified in this study, ‘Family care as a natural responsibility’, can be interpreted as a reflection of the family-centered caregiving culture traditionally rooted in various Asian societies [17,18]. However, this traditional caregiving system in Korean society has gradually declined, making it increasingly difficult to expect adult children to assume the responsibility of supporting their aging parents [17]. Furthermore, there is a growing trend of expecting care from their spouses rather than from their adult children [17,19]. The finding that most caregivers in this study were spouses may reflect these broader sociocultural and generational shifts. Previous studies have shown that when the primary caregiver is a spouse rather than an adult child, lung transplant recipients demonstrate higher long-term survival rates [4]. This suggests that spouses are more actively and consistently involved in caregiving than adult children. Additionally, the presence of three male spousal caregivers in this study reflects a deviation from the traditional Korean cultural norm, which typically assigns caregiving responsibilities to female family members. The spousal relationship, forged through shared experiences of overcoming family crises, may be among the most suitable for caregiving. However, male caregivers may encounter challenges due to their lack of familiarity with nursing care and household management. Furthermore, some caregivers may experience difficulties stemming from financial strain or a lack of alternative caregivers. Therefore, assessing the experiences of individual caregivers and implementing a tailored support system may be necessary.

The experiences described by the participants align with previous findings that caregivers often prioritize the lives of patients over their own, leading lives centered around caregiving responsibilities [6]. Caregivers need time and energy to attend to their health and lives [18]. However, these findings suggest that they find it challenging to care for their needs while being fully engaged in caregiving. Support from other family members or a broader social network is essential for caregivers to recharge and maintain their well-being. Similarly, research on the experience, burden, and coping strategies of caregivers of patients with chronic lung disease [20] and other chronic conditions [21] is gradually increasing. However, social awareness and empathy toward the caregivers of lung transplant patients remain limited. Therefore, raising public awareness about the need for social support and institutional support systems for caregivers is essential.

In this study, caregivers reported a deep sense of fulfillment derived from helping lung transplant patients. This finding aligns with previous research findings [22], suggesting that when caregivers of patients with chronic lung disease participate in rehabilitation programs that contribute to the improvement of patients, it mitigates the burdens of caregiving. The gratitude expressed by the lung transplant recipients is potentially one of the most meaningful sources of fulfillment for caregivers. Programs that promote the sharing of meaningful experiences and mutual appreciation between patients with cancer and their caregivers potentially reduce caregiver stress and improve quality of life [23]. Similarly, programs aimed at fostering and maintaining positive relationships between lung transplant patients and their caregivers may support their emotional well-being and enhance their capacity to provide support. These findings suggest that developing dedicated support programs for caregivers of lung transplant patients benefits the caregivers as well as the patients they care for.

These findings suggest that the caregiving experiences of lung transplant caregivers may vary across sociocultural and racial contexts. Compared to White American caregivers, who reported depression and anger during caregiving [7], the Korean caregivers in this study described fluctuating emotions, including gratitude, sorrow, and anxiety, linked to the patient’s health condition. This implies that Korean caregivers may prioritize the patient’s condition over their well-being. Although direct comparisons are limited due to a lack of studies comparing Korean and White American caregivers, the differences observed align with prior findings, such as that Black caregivers reported greater fulfillment and lower emotional burden than White caregivers, even in more complex situations (dementia or poverty) [24]. Investigating racial differences in caregivers is critical for developing culturally tailored interventions. The Dyadic Cancer Outcomes Framework is a conceptual model designed to understand the health, psychosocial, and relational outcomes of cancer patients and their informal caregivers (e.g., family members, friends, or partners) as an interdependent dyad [25]. This framework may also be applicable to lung transplant recipients and their primary caregivers, offering valuable insights for developing and evaluating interventions that consider their mutual influence.

This study had some limitations. To minimize bias, we used purposive sampling to recruit participants with diverse characteristics including sex, age, caregiving duration, and relationship to patients. However, the single-center design limits generalizability. Future research should incorporate participants from broader sociocultural and ethnic backgrounds to enhance the understanding and applicability of findings on lung transplant caregiving. The cross-sectional design also restricted our ability to examine the dynamic evolution of caregiving experiences over time. Longitudinal or theory-grounded qualitative studies are needed to investigate these trajectories. Although researcher reflexivity and member checking were employed to reduce bias, the complete elimination of subjectivity in data interpretation was unattainable. To mitigate meaning distortion during translation, the following steps were taken. First, bilingual researchers with experience publishing in international journals translated the Korean manuscript into English. Second, two native English-speaking editors (with master’s or doctoral degrees and expertise in academic writing) reviewed the manuscript for terminological and stylistic accuracy. Future research should focus on exploring the specific needs of caregivers of lung transplant patients by developing and implementing tailored nursing interventions that address those needs.

## 5. Conclusions

This study highlights the need for caregivers of lung transplant patients to overcome challenges with gratitude and a sense of purpose, rather than withdrawing from caregiving responsibilities. The strong desire of the patients to live is a crucial motivating factor sustaining the commitment of the caregivers. This finding suggests that involving caregivers in health promotion programs for lung transplant patients may enhance the sense of reward of the caregivers and give greater meaning to their lives. This suggests that developing nursing interventions that support lung transplant patients and their caregivers may be more effective than those targeting caregivers in isolation. The findings of this study may be used to assess the needs of lung transplant recipients and their caregivers in clinical settings and develop interventions that reflect their reciprocal relationship. Furthermore, the study identified the necessity of providing continuous care-related information and education to reduce the stress experienced by caregivers throughout the illness trajectory of lung transplant recipients. It also highlighted the urgent need to expand social care services to alleviate the caregiving burden on caregivers of lung transplant recipients.

## Figures and Tables

**Table 1:** Themes, subthemes, and meaning units on the experience of caregivers of lung transplant recipients.

Themes	Subthemes	Meaning Units
Caring for a patient who has not fully crossed the threshold of death	Watching a loved one in agony at the brink of death	The patient before lung transplantation appeared like a living corpse while being cared for.
		The patient’s physical condition declined to its lowest point.
		The patient showed little likelihood of returning to a normal life.
		An emotional burden was experienced from witnessing the patient’s suffering.
	Pendulum of hope swinging through uncertainty	Relief and joy were experienced following the patient’s recovery after lung transplantation.
		Fear arose that the patient’s condition may deteriorate again.
		Anxiety was felt regarding the patient’s health during the treatment process.
	Holding on for the survival of the patient	Discontinuing care was not considered due to the patient’s strong will to live.
		Caregiving responsibilities for the patient could not be relinquished.
		Concerns about the possibility of re-transplantation were shared with the patient.
Inevitability of care	Family care as a natural responsibility	Care was provided based on the perceived natural duty associated with being a family member.
		Caring for a sick child was regarded as an inherent parental obligation.
		A sense of familial responsibility prompted a commitment to do whatever was necessary.
	Suitable person for patient care	The caregiving situation was viewed as one that required personal involvement.
		Providing care for the patient was considered manageable within the caregiver’s capacity.
		The caregiver was perceived as the most appropriate person to provide care.
	Making every effort to care	Maximum effort was exerted to support the patient while they remained alive.
		A sense of burden was associated with the need to deliver high-quality care.
		Patient care was performed with meticulous attention to detail.
		Feelings of regret were expressed over the inability to provide flawless care.
My life is buried in the life of the patient	Prioritizing the patient over my daily life	Patient care was prioritized over personal well-being and self-care.
		The needs of the patient were placed above personal struggles.
		Daily life became centered around hospital visits and the patient’s condition.
	Patient-centered priority over other family members	Feelings of guilt arose due to the inability to care for other family members.
		Support and understanding from other family members facilitated the caregiving process.
	Becoming a companion in the disease journey of the patient	Awareness developed that health complications could arise even after lung transplantation.
		Continuous presence during the treatment period was maintained out of concern that the patient might face difficulties if left alone.
		Assistance was provided to the patient in managing daily routines including exercise and dietary control.
		Support was offered to help the patient accept and adapt to life under ongoing treatment.
Insights gained from patient care	Enduring and accepting	Composure was maintained to avoid causing anxiety for the patient.
		Extended hospital stays were endured to provide continuous patient care.
		Silent prayers were offered frequently, both day and night.
		Emotional stability was preserved by accepting the patient’s condition.
	Sense of fulfillment	The patient placed significant trust in the caregiver and found comfort in their presence.
		The patient expressed gratitude toward the caregiver.
		Family members conveyed appreciation for the caregiver’s dedication.
		A more positive perspective on life was developed through the caregiving experience.
	Feeling of gratitude	Gratitude was felt for the continued survival of the patient.
		Appreciation was expressed toward the organ donor.
		Gratitude was extended to those who shared caregiving responsibilities including family members and other caregivers.
		Healthcare professionals were acknowledged with sincere appreciation for their efforts.

## Data Availability

Data can be accessed, for example, for verification by reviewers with IRB approval.
